# Enhanced methane production of vinegar residue by response surface methodology (RSM)

**DOI:** 10.1186/s13568-017-0392-3

**Published:** 2017-05-08

**Authors:** Jiayu Feng, Jiyu Zhang, Jiafu Zhang, Yanfeng He, Ruihong Zhang, Chang Chen, Guangqing Liu

**Affiliations:** 1 College of Chemical Engineering, Beijing University of Chemical Technology, 505A Zonghe Building, 15 North 3rd Ring East Road, Beijing, 100029 People’s Republic of China; 2 Department of Biological and Agricultural Engineering, University of California, Davis, CA 95616 USA

**Keywords:** Vinegar residue, Anaerobic digestion, Methane production, Response surface methodology, Efficiency, Interaction

## Abstract

As the by-product of the vinegar production process, a large number of vinegar residue has been abandoned and caused a serious environmental pollution. Anaerobic digestion has been proved to be able to dispose and convert vinegar residue into bioenergy but still need to improve the efficiency. This study applied central composite design of response surface methodology to investigate the influences of feed to inoculum ratio, organic loading, and initial pH on methane production and optimize anaerobic digestion condition. The maximum methane yield of 203.91 mL gVS^−1^ and biodegradability of 46.99% were obtained at feed to inoculum ratio of 0.5, organic loading of 31.49 gVS L^−1^, and initial pH of 7.29, which was considered as the best condition. It has a very significant improvement of 69.48% for methane production and 52.02% for biodegradability compared with our previous study. Additionally, a high methane yield of 182.09 mL gVS^−1^ was obtained at feed to inoculum ratio of 1.5, organic loading of 46.22 gVS L^−1^, and initial pH of 7.32. And it is more appropriate to apply this condition in industrial application owing to the high feed to inoculum ratio and organic loading. Besides, a significant interaction was found between feed to inoculum ratio and organic loading. This study maximized the methane production of vinegar residue and made a good foundation for further study and future industrial application.

## Introduction

**Fig. 1:**
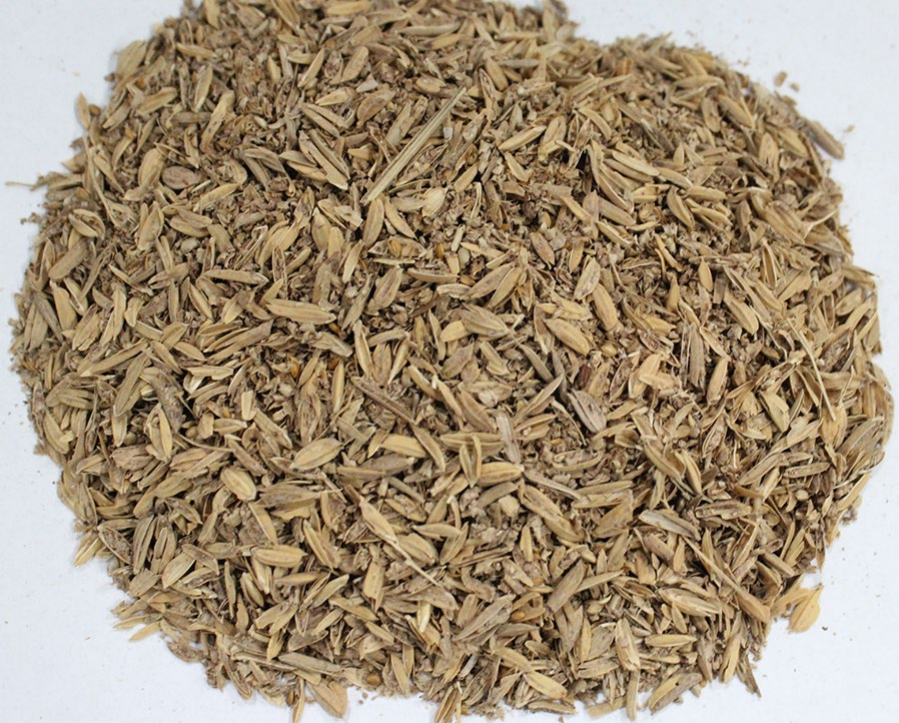
Vinegar residue

Vinegar residue (VR) is a main by-product of vinegar production industry, which is highly developed in China. As the major condiment, the quantity demand of vinegar is very large and every ton of vinegar production could generate 60–70% VR, thus total 3 million tons of VR were produced every year (Chen et al. 2010). VR (shown in Fig. 1) is mainly composed by bran, rice chaff, and other filling materials which are rich in cellulose, hemicellulose, lignin, and pectin (Hou et al. 2011). It also has high acidity and moisture content, thus caused serious environmental pollution in China (Zhong et al. 2012). Traditional treatment methods like landfill and incineration often caused secondary pollution, and it is also a waste of bioresources (Li et al. 2015b). Other methods such as growing plants and feeding to animals are neither economical and effective (Feng et al. 2016). Therefore, it is essential to seek for an alternative method that could deposit VR environmental friendly and effectively, and improve the utilization of VR.

In recent years, anaerobic digestion (AD) has gained more and more attention and obtained great achievements (Yao et al. 2013). Our previous study has proved that VR could be converted by AD but still has great potential of improving methane production (Feng et al. 2013, 2016). AD efficiency is influenced by many different factors such as the feed to inoculum ratio (F/I), organic loading, and initial pH. High F/I ratio and organic loading are preferred by industrial application, however too high value of these two parameters may lead to the accumulation of volatile fatty acids (VFA), thus inhibiting AD. While a lower value of F/I ratio and organic loading could not provide enough nutrition for microorganism growth, thus debasing AD efficiency (Feng et al. 2013; Prashanth et al. 2006). The pH value could also influence the AD process and the appropriate range usually locates from 6.5 to 8.2. The pH can be adjusted automatically by microorganism in a degree and a proper initial pH is easier for microorganism to adjust it into preferred range (Yang et al. 2015). Our previous study showed that VR achieved a methane yield of 120.31 mL gVS^−1^ and a biodegradability (B_d_) of 30.91% at F/I ratio of 1, organic loading of 6 gVS L^−1^ and initial pH of 7. The low methane yield and B_d_ value suggested that there was still more room to improve AD efficiency by optimizing the proper conditions of these three factors and investigating the relationship between them.

Response surface methodology (RSM) is one of the most effective approach for designing experiment, building model, and optimizing condition on responses which influenced by several independent variables (Bezerra et al. 2008; Jiménez et al. 2014; Kang et al. 2016). Compared to the traditional methods, RSM could define not only the influences of independent variables on the responses, but also the interaction between parameters to achieve best system performance (Belwal et al. 2016; Zaroual et al. 2009). The experiment designed by RSM requires fewer tests and shorter time consuming but could obtain a full-experimental design comparison (Khoobbakht et al. 2016). Using RSM, the best operational condition could be found and the interaction between individual factors could be effectively evaluated (Jiménez et al. 2014).

The objectives of this study were to: (1) maximize the AD efficiency of VR by the central composite design (CCD) of RSM; (2) investigate the interaction among F/I ratio, organic loading, and initial pH; (3) evaluate the stability of the AD process by monitoring indicators [finial pH, total ammonia–nitrogen (TAN), total alkalinity (TA), volatile fatty acids (VFA)].

## Materials and methods

### Substrates and inoculum

Vinegar residue was collected from a vinegar factory in Shanxi province, China, and was dried in the room temperature before used. Anaerobic sludge obtained from a biogas plant in Shunyi, Beijing, China was used as inoculum in this study. The sludge was sealed at room temperature and kept for 2 weeks to minimize the background methane production. The characteristics of VR and anaerobic sludge are shown in Table 1.

**Table 1:** Characteristics of VR and inoculum

Parameter	VR	Inoculum
TS (%)^a^	96.60 ± 0.19	14.29 ± 0.10
VS (%)^a^	91.17 ± 0.03	7.56 ± 0.09
VS/TS (%)	94.38 ± 0.15	52.87 ± 0.36
Ash (%)^b^	5.62 ± 0.15	47.13 ± 0.36
Cellulose (%)^b^	22.96 ± 1.43	ND
Hemicellulose (%)^b^	38.90 ± 1.02	ND
Lignin (%)^b^	9.20 ± 0.82	ND
C (%)^b^	46.61 ± 0.02	27.31 ± 0.50
H (%)^b^	6.83 ± 0.03	4.01 ± 0.02
N (%)^b^	2.50 ± 0.05	2.02 ± 0.17
S (%)^b^	0.11 ± 0.01	0.60 ± 0.05
O (%)^b^	37.98 ± 0.09	19.30 ± 0.32
C/N	18.68 ± 0.36	13.57 ± 1.37

*ND* not detectable

^a^As total weight of sample

^b^As TS of sample

### Experiment design by central composite design (CCD) of RSM

The RSM was used to investigate the effects of different factors on methane production of VR and the relationship between them. A five-level-three-factorial experiment was designed by CCD. The variables included F/I ratio (X_1_), organic loading (X_2_), and initial pH (X_3_), and each variable coded at five levels of the high level (+1), the low level (−1), the center point (0) and two outer points corresponding to α (α = 2^k/4^, in this study k = 3, thus α = ±1.68179) (shown in Table 2) (Ahmad et al. 2009; Aslan 2007). Total of 20 runs of experiments were designed instead of the full-experiment, including six center points which represented the estimating of experimental errors and the lack of fit (LOF) and 14 axial points which described the model curvature (shown in Table 3) (Jiménez et al. 2014). Each run of anaerobic digestion was carried out in 500 mL bottle with 200 mL of working volume. The VR, sludge, and deionized water were filled into the bottles and the pH was adjusted according to the design (shown in Table 3). After that, argon was sent into the bottles to replace the atmosphere and then rubber stopper and screw cap were used to seal the bottles to ensure the anaerobic condition. There were three parallel bottles for each condition and three blanks were also used for correction (Feng et al. 2016). All the bottles were placed in an incubator at 37 °C for 45 days and shaken twice a day for about 1 min.

**Table 2:** The level of variables

Variables	Factors	Experimental design (Coded level)				
	(Coded X_i_)	−1.682	−1	0	+1	+1.682
F/I ratio	X_1_	0.5	0.7	1	1.3	1.5
Organic loading (gVS L^−1^)	X_2_	13.18	20	30	40	46.82
Initial pH	X_3_	5.32	6	7	8	8.68

**Table 3:** Methane production and chemical characterization of effluent after AD

F/I ratio (X_1_)	Organic loading (X_2_) (gVS L^−1^)	Initial pH (X_3_)	Methane production		Final pH	TAN (mg L^−1^)	TA (mg CaCO_3_ L^−1^)	VFA (mg L^−1^)	VFA/TA
			Experimental value (mL gVS^−1^)	Predicted value (mL gVS^−1^)					
0.7	20	6	145.27	136.50	6.78 ± 0.08	778.0 ± 38.1	3025.0 ± 35.4	33.99 ± 7.72	0.011
0.7	20	8	155.78	157.47	7.50 ± 0.01	809.0 ± 12.7	7050.0 ± 106.1	31.33 ± 4.38	0.004
0.7	40	6	159.27	152.57	7.24 ± 0.04	904.5 ± 23.3	4962.5 ± 53.0	30.38 ± 0.41	0.013
0.7	40	8	174.79	178.10	7.87 ± 0.01	978.5 ± 14.9	9437.5 ± 88.4	64.86 ± 1.59	0.003
1.3	20	6	92.89	87.04	6.93 ± 0.02	491.0 ± 4.2	3287.5 ± 17.7	70.40 ± 0.13	0.021
1.3	20	8	103.32	107.47	7.39 ± 0.03	562.0 ± 26.9	5087.5 ± 159.1	30.20 ± 3.78	0.006
1.3	40	6	145.35	141.11	6.89 ± 0.08	978.0 ± 18.4	3700.0 ± 106.1	67.86 ± 1.05	0.018
1.3	40	8	159.87	166.10	7.37 ± 0.02	820.5 ± 10.6	5875.0 ± 141.4	52.97 ± 3.24	0.009
0.5	30	7	198.90	203.90	7.69 ± 0.06	910.0 ± 18.4	9337.5 ± 159.1	22.27 ± 1.69	0.002
1.5	30	7	153.62	152.22	7.28 ± 0.03	610.0 ± 35.4	4762.5 ± 53.0	49.32 ± 0.39	0.010
1	13.18	7	102.80	106.79	7.29 ± 0.08	455.0 ± 17.0	3262.5 ± 159.1	16.33 ± 2.10	0.005
1	46.82	7	170.00	169.61	7.50 ± 0.05	901.5 ± 24.8	8562.5 ± 123.7	58.18 ± 5.15	0.007
1	30	5.32	77.59	91.56	6.66 ± 0.05	973.5 ± 7.8	3175.0 ± 70.7	118.24 ± 4.29	0.037
1	30	8.68	140.59	130.22	7.60 ± 0.01	820.0 ± 14.1	8112.5 ± 123.7	24.88 ± 1.77	0.003
1	30	7	168.33	168.60	7.40 ± 0.01	781.5 ± 27.6	5912.5 ± 53.0	28.46 ± 2.31	0.005
1	30	7	160.02	168.60	7.42 ± 0.01	807.5 ± 31.8	5725.0 ± 106.1	30.67 ± 1.68	0.005
1	30	7	175.53	168.60	7.38 ± 0.06	824.0 ± 29.7	5900.0 ± 106.1	27.88 ± 1.92	0.005
1	30	7	178.15	168.60	7.33 ± 0.07	789.0 ± 34.0	5862.5 ± 123.7	25.64 ± 0.69	0.004
1	30	7	162.53	168.60	7.43 ± 0.04	780.0 ± 22.6	6425.0 ± 141.4	30.17 ± 1.90	0.005
1	30	7	167.65	168.60	7.41 ± 0.06	824.0 ± 11.3	5862.5 ± 88.4	31.20 ± 0.53	0.005

*TAN* total ammonia–nitrogen; *TA* total alkalinity; *VFA* volatile fatty acids

### Analytical methods

The values of the total solids (TS), volatile solids (VS), pH, elemental compositions of VR and sludge, the ammonia–nitrogen (TAN), total alkalinity (TA), volatile fatty acids (VFA) of effluent, the headspace pressure, biogas and methane yield and biogas composition were analyzed according to standard methods reported previously (Feng et al. 2016).

### Model analysis

The experimental methane yield (EMY) which represents the highest cumulative methane yield from experiment was fitted to a second-order polynomial model [Eq. (1)] by RSM in order to describe the relationship between the response (methane production) and the variables (F/I ratio, organic loading, and initial pH).

1 $$ {\text{Y}} = {\text{b}}_{0} + \sum\limits_{\text{{i}}} {{\text{b}}_{\text{{i}}} } {\text{X}}_{\text{{i}}} + \sum\limits_{{\text{i}} < {\text{j}}} {\sum\limits_{\text{{j}}} {{\text{b}}_{\text{{ij}}} } } {\text{X}}_{\text{{i}}} {\text{X}}_{\text{{j}}} + \sum\limits_{\text{{i}}} {{\text{b}}_{\text{{ii}}} } {\text{X}}_{\text{{i}}}^{2} $$

where Y means the responses, X represents the variables, and b refers to regression coefficients (b_0_, b_i_, b_ij_ and b_ii_ are the intercept, linear, interaction and quadratic terms, respectively) (Kong et al. 2016; Sumic et al. 2016).

### Theoretical maximum methane yield (MMY) and Biodegradability (B_d_)

MMY of VR was calculated based on elements composition, according to Buswell (Buswell and Mueller 1952) and Chen’s formulas (Shen et al. 2017), shown as Eqs. (2) and (3). B_d_ of VR was determined by EMY and MMY according to Eq. (4) (Ji et al. 2017).

2 $$ \begin{aligned} {\text{C}}_{\text{{n}}} {\text{H}}_{\text{{a}}} {\text{O}}_{\text{{b}}} {\text{N}}_{\text{{c}}}  + \left( {{\text{n}} - \frac{\text{{a}}}{4} - \frac{{\text{b}}}{2} + \frac{3{\text{c}}}{4}} \right) \, {\text{H}}_{2} {\text{O}}\to \,\left( {\frac{{\text{n}}}{2}+ \frac{{\text{a}}}{8} - \frac{{\text{b}}}{4}\, - \,\frac{3{\text{c}}}{8}} \right) \, {\text{CH}}_{4} \hfill \\ + \left( {\frac{{\text{n}}}{2}\, - \,\frac{{\text{a}}}{8} + \frac{{\text{b}}}{4}\, + \,\frac{3{\text{c}}}{8}} \right)\, \, {\text{CO}}_{2} \, + \,{\text{cNH}}_{3} \hfill \\ \end{aligned} $$

3 $$\begin{aligned} {\text{MMY}}\left( {{\text{mL}} \, {\text{gVS}}^{ - 1} } \right) &= \frac{{ 2 2. 4\times 1 0 0 0\times \left( {\frac{\text{n}}{ 2} + \frac{\text{a}}{ 8} - \frac{\text{b}}{ 4} - \frac{{ 3 {\text{c}}}}{ 8}} \right)}}{{ 1 2 {\text{n}} + {\text{a}} + 1 6 {\text{b}} + 1 4 {\text{c}}}} \\ & \quad \times \left( {1 - {\text{Lignin}}\% - {\text{ash}}\% } \right) \end{aligned}$$

4 $$ {\text{B}}_{{\text{d}}} = \frac{{\text{EMY}}}{{\text{MMY}}} \times 100\% $$

### Statistical analysis

Software Design Expert Version 8.05 was used to data calculation, graphing, and modeling. Analysis of variance (ANOVA) was applied to evaluate the adequacy of the model and the statistical significance of the regression coefficients (Kang et al. 2016). The quality of the polynomial model was estimated by using the coefficient of determination (R^2^), model *p*-value, F-test, and lack of fit (LOF) testing (Sumic et al. 2016). The significance of all the terms for variables was tested statistically at level of *p* = 0.05. The best condition for the experiment, the influence of individual variable, and the interaction between variables on the response were described by 2D contour plots and 3D response surface plots (Subha et al. 2015).

## Results

### Interpretation and evaluation of RSM model

The EMY designed by CCD and the predicted values obtained from the software are shown in Table 3. And the influences of the variables on methane production (F/I ratio, organic loading, and initial pH) were fitted to a second-order polynomial model shown as follows [Eq. (5)]:

5 $$ \begin{aligned} {\text{CH}}_{4} ({\text{Y}}) &= 168.60 - 15.36{\text{X}}_{1} + 18.67{\text{X}}_{2} + 11.49{\text{X}}_{3} \hfill \\ & \quad + 9.50{\text{X}}_{1} {\text{X}}_{2} - 0.13{\text{X}}_{1} X_{3} + 1.14{\text{X}}_{2} {\text{X}}_{3} \hfill \\ & \quad + 3.35{\text{X}}_{1}^{2} - 10.75{\text{X}}_{2}^{2} - 20.40{\text{X}}_{3}^{2} \hfill \\ \end{aligned} $$

F-test and *p*-value were applied to evaluate the significance of model and the data obtained by ANOVA are presented in Table 4. The model showed a high F-value of 24.04 and the LOF showed a low F-value of 2.35. The X_1_, X_2_, X_3_, X_1_X_2_, ${\text{X}}_{2}^{2}$, and ${\text{X}}_{3}^{2}$ are significant model terms owning to the lower *p*-values (*p* < 0.05). The R^2^ value of this model was 0.9558 and a coefficient of variation (C.V. %) of 6.13% was obtained.

**Table 4:** ANOVA for response surface quadratic model

Source	Sum of squares	Degree of freedom	Mean squares	F-value	*p*-value prob >F	
Model	18185.28	9	2020.59	24.04	<0.0001	Significant
X_1_-F/I	3223.97	1	3223.97	38.35	0.0001	
X_2_-loading	4762.71	1	4762.71	56.66	<0.0001	
X_3_-pH	1803.34	1	1803.34	21.45	0.0009	
X_1_X_2_	722.00	1	722.00	8.59	0.0150	
X_1_X_3_	0.15	1	0.15	0.00	0.9676	
X_2_X_3_	10.35	1	10.35	0.12	0.7329	
X_1_^2^	161.29	1	161.29	1.92	0.1961	
X_2_^2^	1664.55	1	1664.55	19.80	0.0012	
X_3_^2^	5999.04	1	5999.04	71.36	<0.0001	
Residual	840.62	10	84.06			
Lack of fit	590.02	5	118.00	2.35	0.1845	Not significant
Pure error	250.60	5	50.12			
Cor total	19025.90	19				

Standard deviation	Mean	C.V. %	R-squared	Adjusted R-squared	Predicted R-squared	Adeq precision
9.1700	149.6100	6.1300	0.9558	0.9161	0.7455	18.0260

*C.V. %* coefficient of variation

### Improvement of methane production

Design Expert Software was used to determine the proper range of these variables. The best condition for methane production is F/I ratio of 0.5, organic loading of 31.49 gVS L^−1^ and initial pH of 7.29 and a maximum predicted methane yield of 205.42 mL gVS^−1^ was obtained under this condition. Confirmation experiment was conducted to verify the accuracy of the result at the best condition and a verified value of 203.91 mL gVS^−1^ (46.99% for B_d_) was obtained. On the other hand, a relatively high predicted methane yield of 182.09 mL gVS^−1^ and a B_d_ of 41.96% were obtained at F/I ratio of 1.5, organic loading of 46.22 gVS L^−1^, and initial pH of 7.32.

### Effect of different variables on methane production

**Fig. 2:**
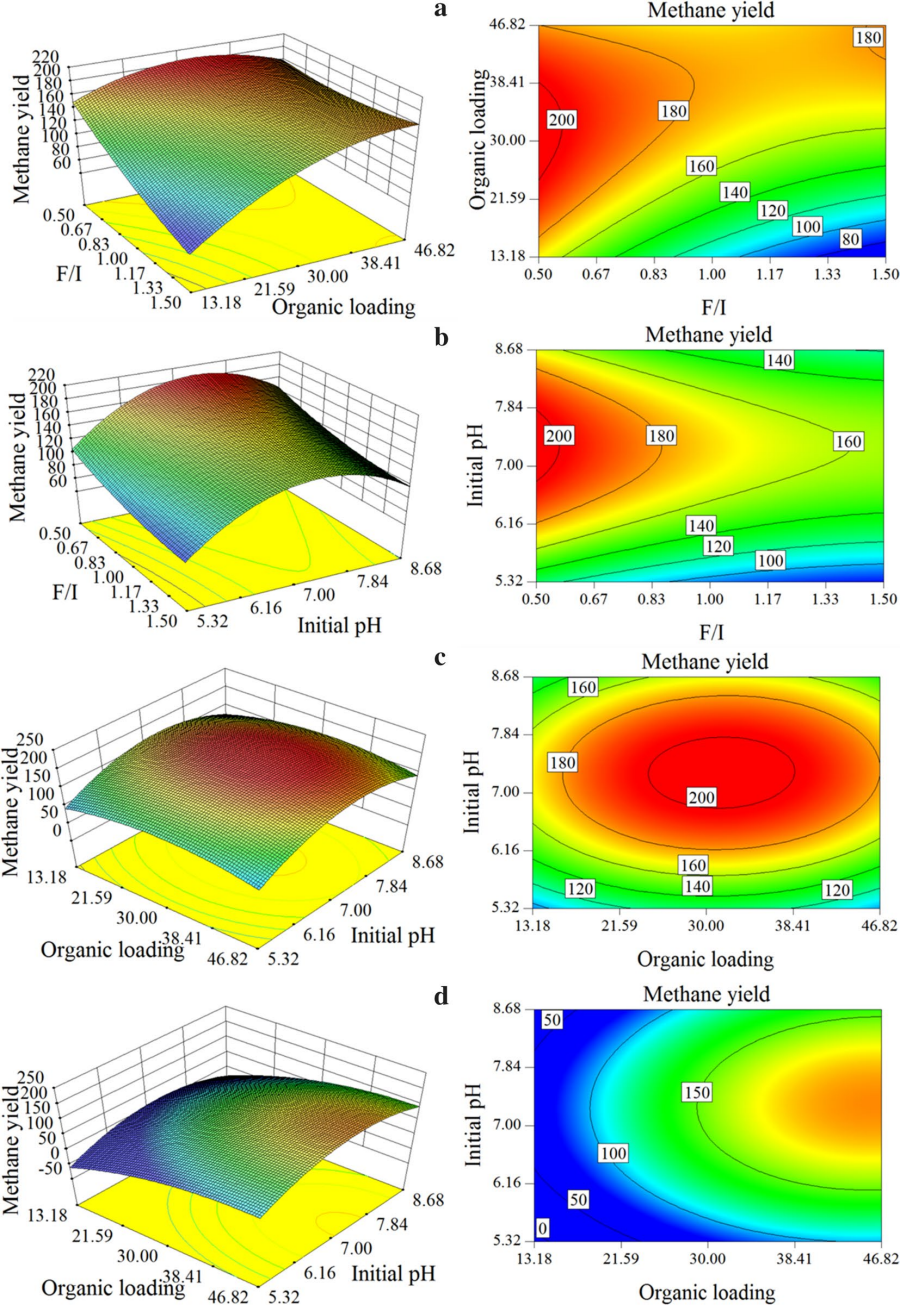
The 3D response surfaces and 2D contour plots for describing the interaction of different variables on methane production

The 3D response surfaces and 2D contour plots which applied to describe the interaction of different variables on methane production are shown in Fig. 2. The F/I ratio, organic loading, and initial pH ranged from 0.5 to 1, 13.18–46.82 gVS L^−1^, and 5.32–8.68, respectively. Figure 2a shows the relationship between F/I ratio and organic loading at initial pH of 7.29. At a relatively higher F/I ratio, methane production increased substantially with organic loading, and a higher predicted methane yield of 182.05 mL gVS^−1^ was obtained at F/I ratio of 1.5, organic loading of 45.87 gVS L^−1^ and initial pH of 7.29. While at a relatively lower F/I ratio, with the increasing of organic loading, methane production increased to a maximum value of 205.42 mL gVS^−1^ at organic loading of 31.49 gVS L^−1^, then decreased gradually. Figure 2b shows the relationship between F/I ratio and initial pH at organic loading of 31.49 gVS L^−1^. With the increasing of F/I ratio, methane production decreased gradually. And with the increasing of initial pH to 7.29, methane production increased firstly, and then decreased gradually. The relationship between organic loading and initial pH at F/I ratio of 0.5 and 1.5 are shown in Fig. 2c, d, respectively. Methane production was first increased and then decreased alone with the increasing of initial pH whatever the F/I ratio and organic loading are. In additionally, it also shows rounded 2D contour plots in Fig. 2c, d.

### Evaluation of AD process stability

Total ammonia–nitrogen (TAN), total alkalinity (TA), volatile fatty acids (VFA), and final pH are the major parameters which could indicate stability of AD, and all of these parameters should be stayed in appropriate range, otherwise the digestion efficiency will be influenced (Li et al. 2015a). The proper range of final pH ranges from 6.5 to 8.2 (Yang et al. 2015) and all the data in this study were located in it, though the initial pH values were different (shown in Table 3). High F/I ratio and organic loading may lead to the accumulation of VFA, while all the groups showed very low value of VFA (lower than 5000 mg L^−1^) (Kim and Kim 2016). The value of VFA/TA is also an important parameter and AD will be stable when VFA/TA stays below 0.4 (Feng et al. 2013). Table 3 shows very low value of VFA/TA for all the groups. The appropriate range of TAN stays below 200 mg L^−1^, and there is also no inhibition for AD when TAN ranges from 200 to 1000 mg L^−1^ (Rajagopal et al. 2013). In this study, all the groups showed proper TAN values lower than 1000 mg L^−1^.

## Discussion

Generally, results from Table 4 were used to evaluate the significant of this model. A higher F-value and a lower *p*-value (*p* < 0.05) mean the significant effect for the model (Ma et al. 2009). The F-value of 24.04 in this study implied the model was significant and there was only a 0.01% (<0.001) chance to obtain a large F-value due to noise (Wang et al. 2012). The lack of fit (LOF) was calculated by pure error and residual error, and the LOF F-value of 2.35 meant that it was not significant relative to the pure error, indicating the model was well fitting (Kong et al. 2016; Wang et al. 2012). The lower *p*-values (*p* < 0.05) of X_1_, X_2_, X_3_, X_1_X_2_, ${\text{X}}_{2}^{2}$, and ${\text{X}}_{3}^{2}$ indicated all the three factors have a significant effect on the response (methane production). The *p*-value of 0.015 for X_1_X_2_ also implied the interaction was happened between F/I ratio and organic loading. The R^2^ value of 0.9558 implied more than 95.58% of the variance was attributable to the variables in methane production (Subha et al. 2015). Coefficient of variation (C.V. %) described the dispersion of the data and represented the accuracy and credibility of the result. A lower C.V. % (<10%) means the smaller variation of the mean value and the C.V. % of 6.13% in this study also implied the significance of the model (Sumic et al. 2016). In general, all the analysis proved that this model was significant and the results obtained by the software were credible.

The verified value of 203.91 mL gVS^−1^ was very close to the predicted value (205.42 mL gVS^−1^), indicating the model was well fitted. Compared with the methane yield of 120.31 mL gVS^−1^ (at F/I ratio of 1, organic loading of 6 gVS L^−1^ and initial pH of 7) and B_d_ of 30.91% in our previous study, it had very significant improvements of 69.48 and 52.02%, respectively. It was obvious that these three factors significantly influenced the AD efficiency and a proper value of them could improve methane production greatly. On the other hand, although the methane yield at F/I ratio of 1.5, organic loading of 46.22 gVS L^−1^, and initial pH of 7.32 was slightly lower than the maximum methane yield (203.91 mL gVS^−1^), the high F/I ratio and organic loading are more preferred in industrial application. In general, it is worthy to do more study on AD of VR under these two conditions in the future.

It could also be found from Fig. 2 that the different variable trend for methane production at different F/I ratio level (shown in Fig. 2a) represented the interaction was significant between F/I ratio and organic loading. The same variable trend for methane production at different initial pH and F/I ratio level (shown in Fig. 2b) implied interaction was not significant between initial pH and F/I ratio. The rounded 2D contour plots (shown in Fig. 2c, d) implied no interaction was generated between initial pH and organic loading (Hatami et al. 2014; Nam and Capareda 2015). In general, interaction was only found between F/I ratio and organic loading, and these two factors influenced the methane production more significantly.

As shown in Table 3, the proper final pH values for all the groups in this study might be because the AD system could adjust pH gradually to some extent and an appropriate initial pH is easier for microorganism to adjust it into proper range. The low value of VFA (lower than 5000 mg L^−1^) and VFA/TA indicated the high organic loading and F/I ratio in this study were acceptable for AD and no inhibition was happened. It is obvious that a high organic loading may lead to relatively higher TAN value while a relatively high F/I ratio could buffer the high TAN contribution of high organic loading. At condition of organic loading of 30 gVS L^−1^, F/I ratio of 0.5, and initial pH of 7, the AD system showed a TAN value of 910 mg L^−1^, which already reached the edge of proper range. If the organic loading increased continually at F/I ratio of 0.5, the TAN value could probably exceed over 1000 mg L^−1^ and AD might be inhibited. That could be a good explanation for the downtrend of methane production when organic loading higher than 31.49 gVS L^−1^ at F/I ratio of 0.5. Similarly, owing to the buffering of high F/I ratio, the TAN value of high organic loading group (more than 40 gVS L^−1^) may not be too high (lower than 1000 mg L^−1^), thus AD efficiency was not inhibited by TAN. In general, the results showed that high organic loading and F/I ratio in this study didn’t influence the stability of AD.

In conclusion, anaerobic digestion efficiency of VR on methane production was improved by using response surface methodology and the interaction among feed to inoculum ratio, organic loading, and initial pH was also investigated in this study. Results showed that the best condition was feed to inoculum ratio of 0.5, organic loading of 31.49 gVS L^−1^, and initial pH of 7.29, which achieved the maximum methane yield of 203.91 mL gVS^−1^ and B_d_ of 46.99%. It increased by 69.48 and 52.02% compared with our previous study, respectively. The feed to inoculum ratio and organic loading showed a significant interaction on methane production. Additionally, high feed to inoculum ratio of 1.5 and organic loading more than 40 gVS L^−1^ were confirmed effectively for anaerobic digestion of vinegar residue and showed a possibility of using them in future industrial application. The findings of this research provided useful information for both fundamental study and industrial application for AD of VR waste, which could not only reduce its pollution, but also facilitate its conversion to renewable energy.

## Abbreviations

AD: anaerobic digestion
ANOVA: analysis of variance
B_d_: biodegradability
CCD: central composite design
C.V. %: coefficient of variation
EMY: experimental methane yield
F/I: feed to inoculum ratio
LOF: lack of fit
MMY: theoretical maximum methane yield
RSM: response surface methodology
TA: total alkalinity
TAN: total ammonia–nitrogen
TS: total solids
VFA: volatile fatty acids
VR: vinegar residue
VS: volatile solids

## References

[CR1] Ahmad AL, Low SC, Shukor SRA, Ismail A (2009). Optimization of membrane performance by thermal-mechanical stretching process using responses surface methodology (RSM). Sep Purif Technol.

[CR2] Aslan N (2007). Application of response surface methodology and central composite rotatable design for modeling the influence of some operating variables of a multi-gravity separator for coal cleaning. Fuel.

[CR3] Belwal T, Dhyani P, Bhatt ID, Rawal RS, Pande V (2016). Optimization extraction conditions for improving phenolic content and antioxidant activity in *Berberis asiatica* fruits using response surface methodology (RSM). Food Chem.

[CR4] Bezerra MA, Santelli RE, Oliveira EP, Villar LS, Escaleira LA (2008). Response surface methodology (RSM) as a tool for optimization in analytical chemistry. Talanta.

[CR5] Buswell AM, Mueller HF (1952). Mechanism of methane fermentation. Ind Eng Chem.

[CR6] Chen X, Wang Z, Ma H, Jiang C, Chang J, Ma X (2010). Situation and prospect of the utilization of vinegar residue. China Brew.

[CR7] Feng L, Li Y, Chen C, Liu X, Xiao X, Ma X, Zhang R, He Y, Liu G (2013). Biochemical methane potential (BMP) of vinegar residue and the influence of feed to inoculum ratios on biogas production. Bioresources.

[CR8] Feng J, Zhang J, Zhang J, He Y, Zhang R, Liu G, Chen C (2016). Influence of steam explosion pretreatment on the anaerobic digestion of vinegar residue. Waste Manag Res.

[CR9] Hatami M, Jafaryar M, Ganji DD, Gorji-Bandpy M (2014). Optimization of finned-tube heat exchangers for diesel exhaust waste heat recovery using CFD and CCD techniques. Int Commun Heat Mass.

[CR10] Hou Y, Lin C, Wang Y, Duan N (2011). Study on anaerobic fermentation characteristics of vinegar residue. Renew Energ Resour.

[CR11] Ji J, Zhang J, Yang L, He Y, Zhang R, Liu G, Chen C (2017). Impact of co-pretreatment of calcium hydroxide and steam explosion on anaerobic digestion efficiency with corn stover. Environ Technol.

[CR12] Jiménez J, Guardia-Puebla Y, Romero-Romero O, Cisneros-Ortiz ME, Guerra G, Morgan-Sagastume JM, Noyola A (2014). Methanogenic activity optimization using the response surface methodology, during the anaerobic co-digestion of agriculture and industrial wastes Microbial community diversity. Biomass Bioenerg.

[CR13] Kang J, Kim S, Moon B (2016). Optimization by response surface methodology of lutein recovery from paprika leaves using accelerated solvent extraction. Food Chem.

[CR14] Khoobbakht G, Najafi G, Karimi M, Akram A (2016). Optimization of operating factors and blended levels of diesel, biodiesel and ethanol fuels to minimize exhaust emissions of diesel engine using response surface methodology. Appl Therm Eng.

[CR15] Kim JR, Kim JY (2016). Feasibility assessment of thermophilic anaerobic digestion process of food waste. J Mater Cycles Waste Manag.

[CR16] Kong Z, Li M, Chen J, Bao Y, Fan B, Francis F, Dai X (2016). Processing factors of triadimefon and triadimenol in barley brewing based on response surface methodology. Food Control.

[CR17] Li C, Champagne P, Anderson BC (2015). Enhanced biogas production from anaerobic co-digestion of municipal wastewater treatment sludge and fat, oil and grease (FOG) by a modified two-stage thermophilic digester system with selected thermo-chemical pre-treatment. Renew Energ.

[CR18] Li L, Feng L, Zhang R, He Y, Wang W, Chen C, Liu G (2015). Anaerobic digestion performance of vinegar residue in continuously stirred tank reactor. Bioresour Technol.

[CR19] Ma H, Liu W, Chen X, Wu Y, Yu Z (2009). Enhanced enzymatic saccharification of rice straw by microwave pretreatment. Bioresour Technol.

[CR20] Nam H, Capareda S (2015). Experimental investigation of torrefaction of two agricultural wastes of different composition using RSM (response surface methodology). Energy.

[CR21] Prashanth S, Kumar P, Mehrotra I (2006). Anaerobic degradability: effect of particulate COD. J Environ Eng Asce.

[CR22] Rajagopal R, Masse DI, Singh G (2013). A critical review on inhibition of anaerobic digestion process by excess ammonia. Bioresour Technol.

[CR23] Shen J, Zhao C, Liu G, Chen C (2017). Enhancing the performance on anaerobic digestion of vinegar residue by sodium hydroxide pretreatment. Waste Biomass Valori.

[CR24] Subha B, Song YC, Woo JH (2015). Optimization of biostimulant for bioremediation of contaminated coastal sediment by response surface methodology (RSM) and evaluation of microbial diversity by pyrosequencing. Mar Pollut Bull.

[CR25] Sumic Z, Vakula A, Tepic A, Cakarevic J, Vitas J, Pavlic B (2016). Modeling and optimization of red currants vacuum drying process by response surface methodology (RSM). Food Chem.

[CR26] Wang X, Yang G, Feng Y, Ren G, Han X (2012). Optimizing feeding composition and carbon-nitrogen ratios for improved methane yield during anaerobic co-digestion of dairy, chicken manure and wheat straw. Bioresour Technol.

[CR27] Yang L, Huang Y, Zhao M, Huang Z, Miao H, Xu Z, Ruan W (2015). Enhancing biogas generation performance from food wastes by high-solids thermophilic anaerobic digestion: effect of pH adjustment. Int Biodeterior Biodegrad.

[CR28] Yao Y, He M, Ren Y, Ma L, Luo Y, Sheng H, Xiang Y, Zhang H, Li Q, An L (2013). Anaerobic digestion of poplar processing residues for methane production after alkaline treatment. Bioresour Technol.

[CR29] Zaroual Z, Chaair H, Essadki AH, El Ass K, Azzi M (2009). Optimizing the removal of trivalent chromium by electrocoagulation using experimental design. Chem Eng J.

[CR30] Zhong M, Wang Y, Yu J, Tian Y, Xu G (2012). Porous carbon from vinegar lees for phenol adsorption. Particuology.

